# Quantitative Evaluation and Selection of Reference Genes for Quantitative RT-PCR in Mouse Acute Pancreatitis

**DOI:** 10.1155/2016/8367063

**Published:** 2016-03-16

**Authors:** Zhaoping Yan, Jinhang Gao, Xiuhe Lv, Wenjuan Yang, Shilei Wen, Huan Tong, Chengwei Tang

**Affiliations:** ^1^Department of Gastroenterology, Affiliated Hospital, Zunyi Medical College, Zunyi, Guizhou 563003, China; ^2^Division of Peptides Related with Human Diseases, State Key Laboratory of Biotherapy of Human Diseases, West China Hospital, Sichuan University, Chengdu, Sichuan 610041, China; ^3^Department of Gastroenterology, West China Hospital, Sichuan University, Chengdu, Sichuan 610041, China

## Abstract

The analysis of differences in gene expression is dependent on normalization using reference genes. However, the expression of many of these reference genes, as evaluated by quantitative RT-PCR, is upregulated in acute pancreatitis, so they cannot be used as the standard for gene expression in this condition. For this reason, we sought to identify a stable reference gene, or a suitable combination, for expression analysis in acute pancreatitis. The expression stability of 10 reference genes (ACTB, GAPDH, 18sRNA, TUBB, B2M, HPRT1, UBC, YWHAZ, EF-1*α*, and RPL-13A) was analyzed using geNorm, NormFinder, and BestKeeper software and evaluated according to variations in the raw Ct values. These reference genes were evaluated using a comprehensive method, which ranked the expression stability of these genes as follows (from most stable to least stable): RPL-13A, YWHAZ > HPRT1 > GAPDH > UBC > EF-1*α* > 18sRNA > B2M > TUBB > ACTB. RPL-13A was the most suitable reference gene, and the combination of RPL-13A and YWHAZ was the most stable group of reference genes in our experiments. The expression levels of ACTB, TUBB, and B2M were found to be significantly upregulated during acute pancreatitis, whereas the expression level of 18sRNA was downregulated. Thus, we recommend the use of RPL-13A or a combination of RPL-13A and YWHAZ for normalization in qRT-PCR analyses of gene expression in mouse models of acute pancreatitis.

## 1. Introduction

Quantitative real-time RT-PCR has become more popular than endpoint RT-PCR for high-throughput and accurate expression profiling of selected genes due to its higher sensitivity, specificity, and broader quantification range [1–3]. However, it is difficult to include the same amount of RNA for samples from different tissues because of differences in cell content or composition of the inflammatory organ [4], differing degrees of RNA degradation [5, 6], and differences in the efficiency of reverse transcription [1–3, 7, 8]. Although a number of common methods are used for normalization, such as adjustment to the input of total RNA [1–3, 7, 8], rRNA [9], or mRNA [10, 11], these are susceptible to impacts of experimental treatments [8, 12]. Thus, the normalization of a reference gene is currently the most accepted method to correct for minor variations in fluctuating samples [1–3, 7–9, 13].

Selection of an appropriate reference gene can reduce differences between specimens to reveal a tangible difference in the specific expression of target genes, and the expression of an ideal reference gene should remain stable across tissues and cells under various experimental conditions [1–3, 7–9, 13]. Recently, a large number of reports have demonstrated that these classic reference genes (e.g., beta-actin ACTB, glyceraldehyde-3-phosphate dehydrogenase GAPDH, and 18s ribosomal RNA 18sRNA) show variations in expression that may be influenced by experimental treatments and are therefore unsuitable for normalization [1–3, 7–11, 13]. Indeed, over 90% of RNA transcription analyses have used only one reference gene [14]. Moreover, a single reference gene may not be applied to standardize the exact amount of RNA input, especially for tissues [9, 15], due to fluctuations in the expression of this reference gene [1–3, 7–9, 13–16]. Ubiquitous reference genes in diverse mammalian expression studies were also not applicable [9, 13, 14, 17]. However, to detect precise changes between different samples of pancreatic tissue, these classical housekeeping genes are not suitable reference genes for qRT-PCR because of the considerable variation in their expression [13, 14, 17]. Thus, the verification of optimal reference genes for qPCR in pancreatic tissues during acute pancreatitis is urgently needed.

But the identification of an optimal reference gene can be a difficult task for the investigator [2]. Also, due to the composition [18, 19] and size [20, 21] of the organ, no constant reference genes have been identified for these studies. The same problem has been reported in pancreatic tissues during acute pancreatitis, when a large number of leucocytes infiltrate into pancreatic tissues and many pancreatic acinar cells undergo necrosis. Because the severity of acute pancreatitis is related not only to activated trypsinogen but also to the amount and type of leucocytes infiltrated, the subsequent necrosis of pancreatic tissues (including RNA degradation) is the result of enzymes released from leucocytes and damaged pancreatic acinar cells. Thus, it was hypothesized that, due to RNA degradation in pancreatic inflammatory tissue and the severity of acute pancreatitis, reference gene expression stability would be affected by the quality of RNA obtained from these tissues [5, 6].

In studies of caerulein-induced murine acute pancreatitis, ACTB [22–28], GAPDH [18, 19, 28–30], 18sRNA [21, 31, 32], beta-2 microglobulin (B2M) [4, 33], and hypoxanthine phosphoribosyltransferase 1 (HPRT1) [34] have been frequently used as reference genes for comparison of mRNA transcription in pancreatic tissue. In fact, because of lower sensitivity and specificity of RT-PCR or northern blot analysis and a large range variation in target mRNA expression, no erroneous conclusions were reported, even if the reference gene expression fluctuated. Jesnowski and colleagues found that ribosomal protein L13A (RPL-13A) is a reliable standard for chronic pancreatitis [35]. And RPL-13A and ubiquitin C (UBC) were shown to be the most stable reference genes in a variety of human cells and tissues [16, 36], while YWHAZ and HPRT1 were the most stable reference genes in partial degradation samples from chronic rhinosinusitis [6].

The current study investigated several common housekeeping genes (ACTB, GAPDH, and 18sRNA), genes commonly used as internal controls, including beta-tubulin (TUBB), UBC, and eukaryotic translational elongation factor 1 alpha (EF-1*α*), and genes that were previously reported as stable reference genes such as B2M [4, 33] and tyrosine 3-monooxygenase/tryptophan 5-monooxygenase activation protein, zeta polypeptide (YWHAZ) [6] during inflammation and infection [37], HPRT1 for cerulein-induced acute pancreatitis mouse model [34], and RPL13A for the patient with acute pancreatitis [35] (see Supplementary Table  3 in Supplementary Material available online at http://dx.doi.org/10.1155/2016/8367063).

This study is the first to perform systematic examination of potential reference genes in pancreatic tissues from mice with caerulein- or LPS-induced acute pancreatitis. We aimed to identify reference genes that were not regulated by caerulein or LPS, and our results should help investigators by providing suitable reference genes for analyses at different time points following the induction of acute pancreatitis.

## 2. Materials and Methods

### 2.1. Animal Model

All experiments were approved by the animal ethics committee of Sichuan University and performed in accordance with established International Guiding Principles for Animal Research. 48 BalB/C mice were fasted from solid food for 12–16 h (but provided with water ad libitum) and were then assigned randomly to either the control group (that received 6 times intraperitoneal natural saline injections), caerulein group (that received 6 times intraperitoneal injections of 50 *μ*g/kg/h caerulein, C9026, Sigma Aldrich) [38], or caerulein + LPS group (that received 6 times intraperitoneal injections of 50 *μ*g/kg/h caerulein, with 10 mg/kg LPS at the last injection,* E. Coli* O111:B4, Sigma Aldrich) [39, 40]. At 1 h, 3 h, 6 h, 9 h, 24 h, and 48 h after the final treatment with caerulein or LPS, six mice (each group) were laparotomized under anesthesia by intraperitoneal administration of 4% chloral hydrate (0.2 mL). The six mice in the control and the caerulein group were treated similarly at 3 h and 6 h after the final injection, respectively. Samples of the pancreas were quickly removed, fresh frozen in liquid nitrogen, and stored at −80°C [41]. Other samples of the pancreas were fixed in 4% v/v neutral phosphate-buffered paraformaldehyde and then embedded in paraffin. Sections were routinely sliced and stained with hematoxylin and eosin for morphologic analysis by light microscopy.

### 2.2. RNA Isolation and Reverse Transcription

Approximately 20 mg of pancreatic tissue was ground into tissue powder in liquid nitrogen, and RNA was then extracted with RNAiso reagent (Takara). Briefly, after RNA was pelleted by centrifugation (10000 g for 15 min at 4°C), the pellet was washed in 75% v/v ethanol twice. The amount of RNA was determined spectrophotometrically by using Gene Quant100 (GE, USA) instrument; and the integrity was verified by Gold view staining of 18s and 28s rRNA bands on a 1% denaturing agarose gel. After incubation with DNase I (Roche) at 37°C for 30 minutes, RNA (3 *μ*g) was reverse-transcribed with random primers using the RevertAid First Stand cDNA Synthesis Kit (Thermo Scientific, 1622).

### 2.3. Quantitative RT-PCR

Quantitative RT-PCR analysis was performed using the primers shown in Table 1. And qRT-PCR was performed with the BioRad CFX96 Sequence Detection System by using the SYBR Green PCR Master Mix (Qiagen). The PCR reaction consisted of 10 *μ*L of SYBR Green PCR Master Mix, 1 *μ*L of forward and reverse primers (4 *μ*M), and 1 *μ*L of template cDNA in a total volume of 20 *μ*L. Cycling was performed using the conditions specified in the BioRad CFX96 Manager Version Software 3.1, consisting of 5 min at 95°C, 10 sec at 95°C, and 30 sec at 60°C, followed by 40 rounds of 10 sec at 95°C and 30 sec at 60°C. Triplicates of all reactions were analyzed. Each assay also included three blanks.

### 2.4. Evaluation of the Stability of Candidate Reference Genes

To evaluate the stability of candidate reference genes expressed as Ct values, we used the software provided by BioRad (version 3.1), which was consistent with the statistical algorithm of geNorm. This program calculated the* M* value of each candidate gene pairwise. After stepwise exclusion of the gene with the highest* M* value, the remaining genes were recalculated for* M* values in turn. In the end, the two genes with the lowest average* M* value were accepted. This iterative process was enabled to rank these candidate genes based on their stability of expression [16].

NormFinder is another Excel-Based Visual Basic for Applications used to identify the optimal reference gene [42].

BestKeeper was used as an Excel-based software tool to determine the best suitable reference genes using pairwise correlation analysis of candidate reference genes [43].

The comparative ΔCt method was performed to assess the most stable reference genes by comparing relative expression within each tissue sample or each treatment [44].

The stability of a gene was measured by the mean of standard deviation values derived from comparisons among reference genes [45, 46].

### 2.5. Statistical Analysis

Comparison of variances for the expression of distinct genes was used to analyze the significance of differences between control and treated groups using one-way analysis of variance (ANOVA) (GraphPad Prisms Software version 6, San Diego, CA, USA). A *p* value <0.05 was considered statistically significant.

## 3. Results

### 3.1. Acute Pancreatitis Induced by Caerulein or LPS

**Figure 1:**
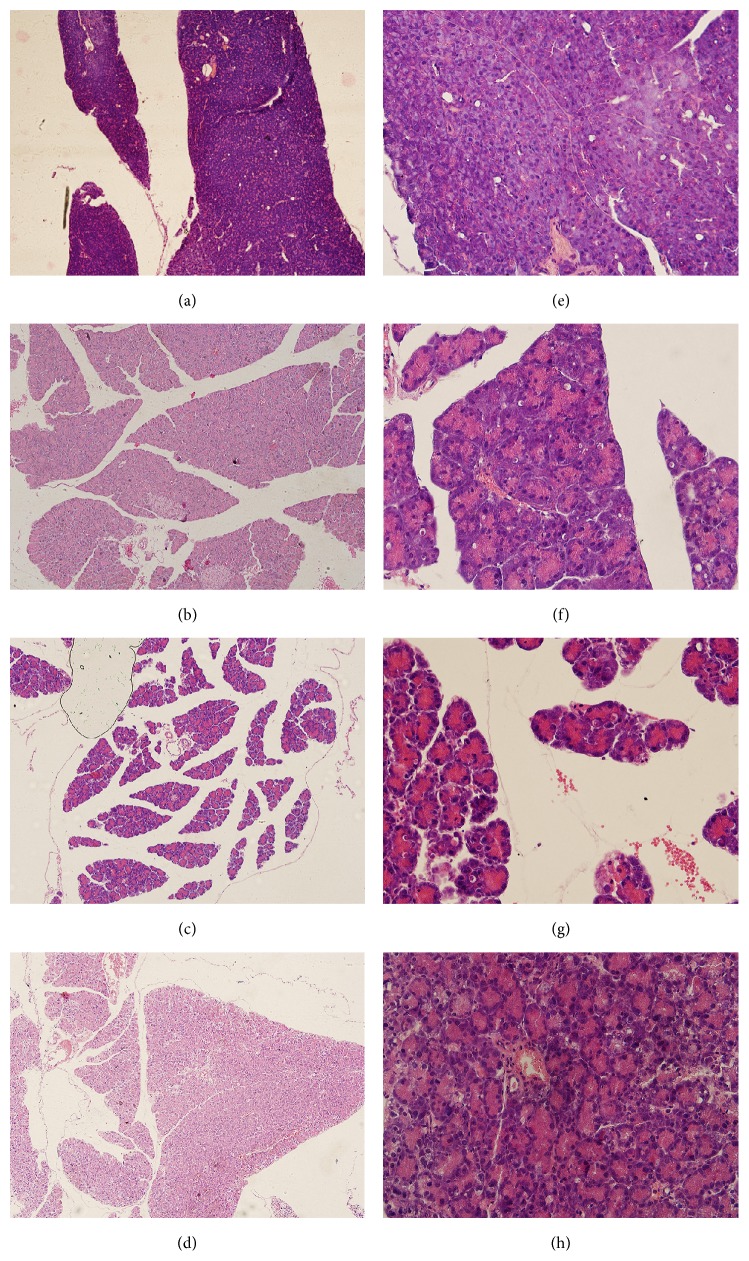
Histological changes in the pancreas of treated mice. HE stain, from (a) to (d): ×100; from (e) to (h): ×400. (a/e) Control: normal pancreas; (b/f) MAP 3 h; (c/g) SAP 3 h; (d/h) SAP 48 h.

All mice given peritoneal injections of caerulein and/or LPS survived. By visual inspection, the volume of the pancreas in the treatment groups was larger than that in the control group, of which treated was pale. Glandular swelling, spacing broadening, necrosis of acinar cells, infiltration of inflammatory cells, and hemorrhage were observed by light microscopy in acute pancreatitis mice. No pathological changes were observed in the control mice (Figure 1).

### 3.2. Total RNA Quality

Cut-off values of >1.8 (OD260/280) and >2.0 (OD260/230) were used as quality measures of total RNA samples. RNA integrity analysis, using denaturing gel electrophoresis, showed distinct and sharp 28s and 18s RNA bands. To prove contaminating genomic DNA, untranscribed (RT-minus control) RNA samples were used.

### 3.3. Expression Profiles of Candidate Reference Genes

The transcript abundance of commonly used reference genes was analyzed in pancreatic samples by direct comparison of their Ct values, with a precondition of an equal quantity of total RNA and RNA degradation. The data of qRT-PCR reactions (Table 1) was in line with MIQE guidelines [47]. And melting curve analysis revealed that all primer pairs amplified a single PCR product.  Figure 3 shows the mean Ct values of 10 selected genes. The highest variation was observed for ACTB, while RPL-13A showed the lowest dispersion, and YWHAZ showed higher dispersion than RPL-13A only.

### 3.4. Caerulein and LPS Affect the Expression Level of Reference Genes

**Figure 2:**
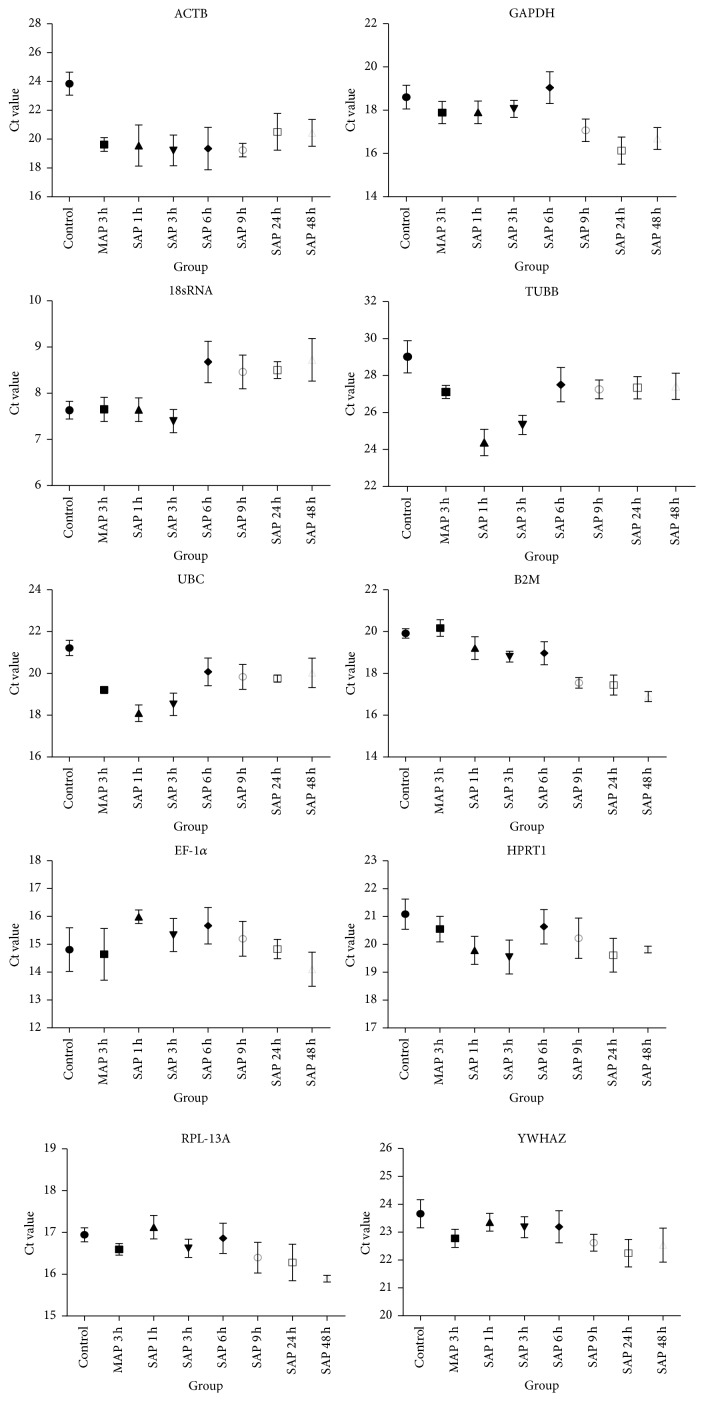
Effect of caerulein or LPS stimulation on reference gene expression.

Pancreatic mRNA expression differences were investigated for 10 reference genes in conditions without stimulation or following stimulation with caerulein or LPS (Figure 2). The mRNA expression of candidate reference genes is indicated by the raw Ct value at discrete time points after caerulein or LPS peritoneal injection.

Distributions of raw Ct values for each reference gene were visualized as column-means and error bars. Column-means show the distributions of raw Ct values (arithmetic means ± SD, *n* = 4–6) for each of the genes tested (MAP 3 h: mild acute pancreatitis induced by caerulein, at 3 h after the last caerulein injection; SAP 1 h: severe acute pancreatitis induced by caerulein and LPS, at 1 h after the last caerulein and LPS injection; SAP 3 h: 3 h after the last caerulein and LPS injection; SAP 6 h: 6 h after the last caerulein and LPS injection; SAP 9 h: 9 h after the last caerulein and LPS injection; SAP 24 h: 24 h after the last caerulein and LPS injection; SAP 48 h: 48 h after the last caerulein and LPS injection).

### 3.5. Stability of Reference Gene Expression

**Figure 3:**
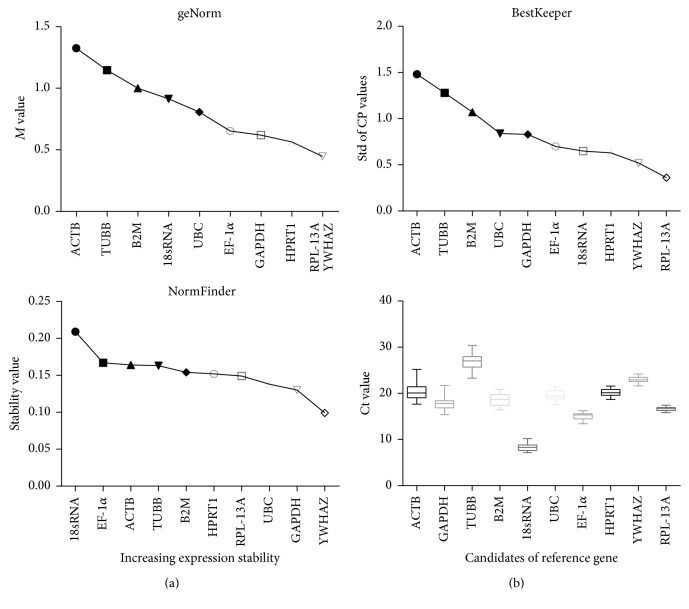
Expression stability values of candidate reference genes during acute pancreatitis.

Reference genes were then ranked by stepwise exclusion of the gene with the highest* M* value (Figure 3). Based on these* M* values, the most stable reference genes with the lowest* M* values were RPL-13A and YWHAZ, while the most unstable was ACTB. According to the algorithm of NormFinder, the most stable reference gene was YWHAZ, and the best combination of two reference genes was YWHAZ and GAPDH (Figure 3). The data obtained with BestKeeper software showed that RPL-13A was the optimal reference gene, with RPL-13A and YWHAZ as the most appropriate combination, which was similar to the results from the geNorm algorithm (Figure 3).

Average expression stability values of candidate reference genes using geNorm, NormFinder, and BestKeeper analysis were performed across all samples. The gene stability value is based on the average pairwise variation between all tested reference genes. The expression stability of genes indicates the least stable (Figure 3(a)) to the most stable (Figure 3(b)) gene.

Distribution of qRT-PCR quantification cycle values for candidate reference genes was visualized as column means and error bars (Figure 3(b)). The mRNA expression of 10 candidate reference genes is shown for mice in the control and experiment groups treated with caerulein and/or LPS. Box and whiskers represent the variance in expression of a particular gene among the specimens. The values are expressed as the mean ± SD.

### 3.6. Final Ranking of Candidate Reference Genes

Considering the ranking results from the four algorithms, we obtained a comprehensive ranking of reference genes (Table 2) [45, 46]. The three most stable reference genes were RPL-13A, YWHAZ, and HPRT1, and the least stable reference genes were ACTB, TUBB, and B2M.

## 4. Discussion

In this study, we evaluated 10 reference genes using qPCR in 45 pancreatic tissues from mice with caerulein- or LPS-induced acute pancreatitis.

The level of ACTB mRNA in pancreatic tissues stimulated by caerulein or LPS was increased significantly compared to that in the control group. Yuan and colleagues found that the level of ACTB mRNA in rat pancreatic tissues increased continuously after caerulein infusion, and immunostaining for ACTB was observed along the membrane of large cytoplasmic vacuoles in pancreatic acinar cells [48]. Furthermore, repeated experimental data suggest that ACTB is not a suitable reference gene under certain conditions [49–51]. Unfortunately, many researchers have ignored these data and misinterpreted the increased level of ACTB as differences among samples or RNA inputs [22, 24–28].

We observed a significant difference among TUBB mRNA expressions in pancreatic tissues from the control group MAP and SAP group. Similar to ACTB, TUBB was not an appropriate reference gene because its expression fluctuated with the pathophysiological process of acute pancreatitis [52].

GAPDH mRNA expression in pancreatic tissue did not vary in animals with MAP induced by caerulein only and in those in the early phase of SAP following treatment with caerulein and LPS, although this expression increased in the later phase (SAP 9 h, SAP 24 h, and SAP 48 h). This result is consistent with previous reports [53, 54] and draws a distinction between Yuan et al.'s results, where GAPDH mRNA increased after 6 h of caerulein treatment [48]. Although GAPDH is a suitable reference gene in mouse caerulein-induced acute pancreatitis [18, 19, 29, 30] and during the early phase of mouse SAP induced by caerulein and LPS, the variation in GAPDH mRNA expression should not be ignored, which probably results from glucose metabolism disturbance in the later phase of the illness [54].

18sRNA expression in pancreatic tissues did not vary during MAP induced by caerulein only and during the early phase (within 3 h after LPS injection) of SAP induced by caerulein and LPS, which is consistent with Yamada et al.'s reports [20]. Thus, with very high abundance and resistance to degradation compared to other genes [5], 18sRNA may have lower variance as a reference gene in pancreatic tissue. However, 18sRNA expression in pancreatic tissue was downregulated in the later phase (6–48 h after LPS injection), which is consistent with the lower level of 18sRNA reported in pancreatic carcinoma compared to normal pancreatic tissue [51]. Similar to GAPDH, 18sRNA may represent a suitable reference gene in mouse caerulein-induced acute pancreatitis [21, 31, 32], or the early phase (within 3 h after LPS injection) of mouse acute pancreatitis by caerulein and LPS. However, 18sRNA was not a suitable reference gene for analyses of the later phase of SAP.

The mRNA expression of B2M and HPRT1 in pancreatic tissues remained constant between the control group and MAP group, which indicated that B2M and HPRT1 are appropriate reference genes for caerulein-induced acute pancreatitis [4, 34]. However, in the SAP groups, the level of B2M mRNA was increased and especially high at discrete time points after LPS injection (the level of SAP 9 h was higher than that of SAP 6 h). Thus, B2M was not a suitable reference gene for SAP. Because of its enriched expression in immunocytes, variation in B2M mRNA expression may correspond to the infiltration of leukocytes into pancreatic tissues at different stages of acute pancreatitis, according to the cell composition in the inflammatory tissue [55, 56].

RPL13A was shown to be the most stable reference gene in a variety of human cells and tissues [16, 35, 36], verified in other experiments [57–59]. Nevertheless, the level of RPL-13A mRNA expression among groups was unstable, even if such changes were small. Due to the unavoidable degradation of RNA by enzymes in pancreatic tissue during acute pancreatitis, YWHAZ should serve as a suitable reference gene. However, the level of YWHAZ mRNA expression showed slight fluctuations among and within groups.

Software analysis using the geNorm algorithm identified RPL-13A and YWHAZ as the most stable genes, followed by HPRT1 and GAPDH. The ranking order presented was similar to that provided using BestKeeper and based on the variation in raw Ct values, except for GAPDH showing a lower rank. However, the NormFinder analysis identified YWHAZ as the best reference gene with the highest expression stability, along with GAPDH, UBC, and RPL-13A. Interestingly, with the top rank in other algorithms, RPL-13A was ranked as the fourth best reference gene by NormFinder. Thus, the ranking order of RPL-13A by NormFinder was not consistent with the results of other algorithms. And there was no obvious modification of the ranking order of YWHAZ. So, based on the combined results of all algorithms, the comprehensive ranking presented RPL-13A and YWHAZ as the optimal reference genes. These reference genes were evaluated using a comprehensive method, which ranked the expression stability of these genes as follows (from most stable to least stable): RPL-13A, YWHAZ > HPRT1 > GAPDH > UBC > EF-1*α* > 18sRNA > B2M > TUBB > ACTB. RPL-13A was the most suitable reference gene, and the combination of RPL-13A and YWHAZ was the most stable group of reference genes in our experiments.

## 5. Conclusions

As discussed above, we observed variations in the expression of commonly used reference genes in response to injection with caerulein and LPS, and no absolutely stable reference gene was identified in pancreatic tissue from animals with acute pancreatitis. Nevertheless, RPL-13A was the most suitable reference gene, while RPL-13A and YWHAZ were identified as the best combination of reference genes.

## Supplementary Material

In the supplementary we provided that the reason why the current reference genes were chosen for candidates and there were specific characters in the pathophysiologic mechnism of severe acute pancreatitis mice models, and that some descriptions about the mice models by caerulein and LPS, some descriptions about qPCR, the histopathologic scoring criteria, the integrity of RNA samples verified by RNA electrophrosis and a table listed about the full names and functions of reference genes.

## Figures and Tables

**Table 1:** Primer sequences for reference genes.

Gene symbol	Primer	NCBI accession numbers	PCR efficiency	Amplicon length
ACTB	F: TGACGTTGACATCCGTAAAG	NM_007393.3	90.9%	143 bp
	R: GAGGAGCAATGATCTTGATCT			

GAPDH	F: GGTGAAGGTCGGTGTGAACG	NM_001289726.1	101.9%	233 bp
	R: CTCGCTCCTGGAAGATGGTG			

18sRNA	F: GTAACCCGTTGAACCCCATT	NR_003278.3	95.1%	151 bp
	R: CCATCCAATCGGTAGTAGCG			

B2M	F: GCTATCCAGAAAACCCCTCAA	NM_009735.3	96.2%	300 bp
	R: CATGTCTCGATCCCAGTAGACGGT			

RPL13A	F: GAGGTCGGGTGGAAGTACCA	NM_009438.5	95.2%	71 bp
	R: TGCATCTTGGCCTTTTCCTT			

EF1*α*	F: CTGAACCATCCAGGCCAAAT	NM_010106.2	98.0%	60 bp
	R: GGCTGTGTGACAATCCAG			

UBC	F: GCCCAGTGTTACCACCAAGA	NM_019639.4	98.6%	104 bp
	R: CCCATCACACCCAAGAACA			

HPRT1	F: CCAGCGTCGTGATTAGCG	NM_013556.2	96.3%	222 bp
	R: CCAGCAGGTCAGCAAAGAAC			

YWHAZ	F: CAGTAGATGGAGAAAGATTTGC	NM_001253807	97.4%	92 bp
	R: GGGACAATTAGGGAAGTAAGT			

TUBB	F: CGGCAACTATGTAGGGGACT	NM_178014.3	96.7%	194 bp
	R: CAGCACCACTCTGACCAAAG			

**Table 2:** Comprehensive ranking of reference genes.

Ranking order	geNorm	NormFinder	BestKeeper	Variation of raw Ct values	Comprehensive ranking (mean rank value)
1	RPL-13A YWHAZ	YWHAZ	RPL-13A	RPL-13A	RPL-13A YWHAZ 1.414

2	HPRT1	GAPDH	YWHAZ	YWHAZ	HPRT1 3.080

3	GAPDH	UBC	HPRT1	HPRT1	GAPDH 4.242

4	EF-1*α*	RPL-13A	18sRNA	EF-1*α*	UBC 4.786

5	UBC	HPRT1	EF-1*α*	UBC	EF-1*α* 5.180

6	18sRNA	B2M	GAPDH	18sRNA	18sRNA 6.160

7	B2M	TUBB	UBC	B2M	B2M 6.694

8	TUBB	ACTB	B2M	TUBB	TUBB 7.969

9	ACTB	EF-1*α*	TUBB	GAPDH	ACTB 9.212

10	—	18sRNA	ACTB	ACTB	—

## Abbreviations

MAP: Mild acute pancreatitis
SAP: Severe acute pancreatitis
LPS: Lipopolysaccharide
*μ*g: Microgravity
qRT-PCR: Quantitative real-time reverse-transcription PCR
Ct: Cycle threshold
SD: Standard deviation
ΔCt: Variation of cycle threshold
ΔΔCt value: Variation of cycle threshold normalized by reference gene
MIQE: Minimum information for publication of quantitative real-time PCR experiments
*M* value: Average stability measured by statistical algorithm of geNorm.
