# Corrigendum to “Quantitative Evaluation and Selection of Reference Genes for Quantitative RT-PCR in Mouse Acute Pancreatitis”

**DOI:** 10.1155/2017/3498537

**Published:** 2017-04-13

**Authors:** Zhaoping Yan, Jinhang Gao, Xiuhe Lv, Wenjuan Yang, Shilei Wen, Huan Tong, Chengwei Tang

**Affiliations:** ^1^Division of Peptides Related with Human Diseases, State Key Laboratory of Biotherapy of Human Diseases, West China Hospital, Sichuan University, Chengdu, Sichuan 610041, China; ^2^Department of Gastroenterology, West China Hospital, Sichuan University, Chengdu, Sichuan 610041, China

In the article titled “Quantitative Evaluation and Selection of Reference Genes for Quantitative RT-PCR in Mouse Acute Pancreatitis” [1], the affiliation of the first author should be “Division of Peptides Related with Human Diseases, State Key Laboratory of Biotherapy of Human Diseases, West China Hospital, Sichuan University, Chengdu, Sichuan 610041, China” only. The corrected authors' list and affiliations are shown above.
