# Supplementary material for: Quantitative Evaluation and Selection of Reference Genes for Quantitative RT-PCR in Mouse Acute Pancreatitis
Source: Biomed Res Int. 2016 Mar 16;2016:8367063. doi: 10.1155/2016/8367063 (PMC4812220; doi:10.1155/2016/8367063)
Supplement: Supplementary file 1 — In the supplementary we provided that the reason why the current reference genes were chosen for candidates and there were specific characters in the pathophysiologic mechnism of severe acute pancreatitis mice models, and that some descriptions about the mice models by caerulein and LPS, some descriptions about qPCR, the histopathologic scoring criteria, the integrity of RNA samples verified by RNA electrophrosis and a table listed about the full names and functions of reference genes. [file 8367063.f1.pdf]

Supplement Table 3 Reference genes evaluated in this study and their function.

Gene symbol	Gene name	Brief description
ACTB	Beta-actin	Cytoskeletal structural protein
GAPDH	Glyceraldehyde-3-phosphate dehydrogenase	Enzyme involved in glycolysis
18s RNA	18s Ribosomal RNA	Component of a ribosomal subunit
B2M	Beta-2 microglobulin	Beta-chain of major histocompatibility complex class I molecules
RPL13A	Ribosomal protein L13A	Ingredient of the large 60s ribosomal subunit
EF1α	Eukaryotic translational elongation factor 1 alpha	Involed in translation
UBC	Ubiquitin C	Protein degradation
HPRT1	Hypoxanthine phosphoribosyltransferase 1	Glycosyltransferase, purine synthesis in salvage pathway
YWHAZ	Tyrosine 3-monooxygenase/tryptophan 5-monooxygenase activation protein, zeta polypeptide	Phosphorylated signaling molecules
